# Supplementary material for: EnzML: multi-label prediction of enzyme classes using InterPro signatures
Source: BMC Bioinformatics. 2012 Apr 25;13:61. doi: 10.1186/1471-2105-13-61 (PMC3483700; doi:10.1186/1471-2105-13-61)
Supplement: Addtional file 5 — The Java code to format the data files, evaluate and predict. The file enzml_java_code.tar.gz contains the Java code used to format database data to ARFF and XML formats, to execute cross and train-test (jackknife) evaluations and to record evaluation results to database. More information is included in the readme.txt file and the Javadoc files. The code can be used with a MySQL database. To use a different database software, other JDBC drivers might be required. [file 1471-2105-13-61-S5.gz › java_code/enzml2011/doc/uk/ac/ed/inf/taweka/test/weka/DataSetManagerTest.html]

DataSetManagerTest


---


|  |  |  |
| --- | --- | --- |
| **Overview** \| **Package** \| **Class** \| **Use** \| **Tree** \| **Deprecated** \| **Index** \| **Help** | |  |
| **PREV CLASS**   **NEXT CLASS** | **FRAMES**    **NO FRAMES**     **All Classes** |  |
| SUMMARY: NESTED \| FIELD \| CONSTR \| METHOD | DETAIL: FIELD \| CONSTR \| METHOD |  |


---


## uk.ac.ed.inf.taweka.test.weka Class DataSetManagerTest

```
java.lang.Object
  junit.framework.Assert
      junit.framework.TestCase
          uk.ac.ed.inf.taweka.test.weka.DataSetManagerTest
```

**All Implemented Interfaces:**: junit.framework.Test

---

``` public class DataSetManagerTest extends junit.framework.TestCase ```

Class

**Version:**
:   8 Sep 2010

**Author:**
:   Luna De Ferrari luna.deferrari-at-ed.ac.uk

---

| **Field Summary** | |
| --- | --- |
| `static java.lang.String` | `arffInstance1` |
| `static java.lang.String` | `arffInstance1b` |
| `static java.lang.String` | `arffInstance2` |
| `static java.lang.String` | `arffInstance4` |
| `static java.lang.String` | `ATT1` |
| `static java.lang.String` | `ATT1_TO_STRING` |
| `static java.lang.String` | `ATT2` |
| `static java.lang.String` | `ATT3` |
| `static java.lang.String` | `ATT3_TO_STRING` |
| `static java.lang.String` | `ATT4` |
| `static java.lang.String` | `ATT4_TO_STRING` |
| `static java.lang.String` | `CLASS_TO_STRING` |
| `static java.lang.String` | `CLASS1` |
| `static java.lang.String` | `CLASS2` |
| `static java.lang.String` | `CLASS3` |
| `static java.lang.String` | `INST1` |
| `static java.lang.String` | `INST2` |
| `static java.lang.String` | `INST3` |
| `static java.lang.String` | `INST4` |
| `static java.lang.String` | `m_arff` |
| `static java.lang.String` | `NAME` |


**Constructor Summary**

`DataSetManagerTest()`


| **Method Summary** | |
| --- | --- |
| `static java.util.Vector<java.lang.String>` | `attributes()`             Data: ATT1 ATT2 ATT3 CLASS instA y n n 1 instB y y n 2 instC n n y 2 |
| `static java.util.Vector<java.lang.String>` | `classValues()` |
| `static DataSetManager` | `getDataSetManager()` |
| `static DataSetManager` | `getDataSetWithNoXmlProperty()` |
| `static java.lang.String` | `getExpectedArff()` |
| `static java.util.Vector<java.lang.String>` | `instances()` |
| `void` | `setUp()` |
| `void` | `testGetArff()` |
| `void` | `testGetDataSetName()` |

**Methods inherited from class junit.framework.TestCase**

`countTestCases, getName, run, run, runBare, setName, toString`

**Methods inherited from class junit.framework.Assert**

`assertEquals, assertEquals, assertEquals, assertEquals, assertEquals, assertEquals, assertEquals, assertEquals, assertEquals, assertEquals, assertEquals, assertEquals, assertEquals, assertEquals, assertEquals, assertEquals, assertEquals, assertEquals, assertEquals, assertEquals, assertFalse, assertFalse, assertNotNull, assertNotNull, assertNotSame, assertNotSame, assertNull, assertNull, assertSame, assertSame, assertTrue, assertTrue, fail, fail, failNotEquals, failNotSame, failSame, format`

**Methods inherited from class java.lang.Object**

`equals, getClass, hashCode, notify, notifyAll, wait, wait, wait`

**Field Detail**

### arffInstance1

```
public static final java.lang.String arffInstance1
```

**See Also:**: Constant Field Values

---


### arffInstance1b

```
public static final java.lang.String arffInstance1b
```

**See Also:**: Constant Field Values

---


### arffInstance2

```
public static final java.lang.String arffInstance2
```

**See Also:**: Constant Field Values

---


### arffInstance4

```
public static final java.lang.String arffInstance4
```

**See Also:**: Constant Field Values

---


### ATT1

```
public static final java.lang.String ATT1
```

**See Also:**: Constant Field Values

---


### ATT1\_TO\_STRING

```
public static final java.lang.String ATT1_TO_STRING
```

**See Also:**: Constant Field Values

---


### ATT2

```
public static final java.lang.String ATT2
```

**See Also:**: Constant Field Values

---


### ATT3

```
public static final java.lang.String ATT3
```

**See Also:**: Constant Field Values

---


### ATT3\_TO\_STRING

```
public static final java.lang.String ATT3_TO_STRING
```

**See Also:**: Constant Field Values

---


### ATT4

```
public static final java.lang.String ATT4
```

**See Also:**: Constant Field Values

---


### ATT4\_TO\_STRING

```
public static final java.lang.String ATT4_TO_STRING
```

**See Also:**: Constant Field Values

---


### CLASS\_TO\_STRING

```
public static final java.lang.String CLASS_TO_STRING
```

**See Also:**: Constant Field Values

---


### CLASS1

```
public static final java.lang.String CLASS1
```

**See Also:**: Constant Field Values

---


### CLASS2

```
public static final java.lang.String CLASS2
```

**See Also:**: Constant Field Values

---


### CLASS3

```
public static final java.lang.String CLASS3
```

**See Also:**: Constant Field Values

---


### INST1

```
public static final java.lang.String INST1
```

**See Also:**: Constant Field Values

---


### INST2

```
public static final java.lang.String INST2
```

**See Also:**: Constant Field Values

---


### INST3

```
public static final java.lang.String INST3
```

**See Also:**: Constant Field Values

---


### INST4

```
public static final java.lang.String INST4
```

**See Also:**: Constant Field Values

---


### m\_arff

```
public static java.lang.String m_arff
```

---


### NAME

```
public static final java.lang.String NAME
```

**See Also:**: Constant Field Values


**Constructor Detail**

### DataSetManagerTest

```
public DataSetManagerTest()
```


**Method Detail**

### setUp

```
public void setUp()
           throws java.lang.Exception
```

:   **Overrides:**: `setUp` in class `junit.framework.TestCase`
:   **Throws:**: `java.lang.Exception`

---


### testGetArff

```
public void testGetArff()
```

---


### testGetDataSetName

```
public void testGetDataSetName()
```

---


### attributes

```
public static java.util.Vector<java.lang.String> attributes()
```

:   Data: ATT1 ATT2 ATT3 CLASS instA y n n 1 instB y y n 2 instC n n y 2

---


### classValues

```
public static java.util.Vector<java.lang.String> classValues()
```

---


### getDataSetManager

```
public static DataSetManager getDataSetManager()
```

---


### getDataSetWithNoXmlProperty

```
public static DataSetManager getDataSetWithNoXmlProperty()
```

---


### getExpectedArff

```
public static java.lang.String getExpectedArff()
```

---


### instances

```
public static java.util.Vector<java.lang.String> instances()
```


---


|  |  |  |
| --- | --- | --- |
| **Overview** \| **Package** \| **Class** \| **Use** \| **Tree** \| **Deprecated** \| **Index** \| **Help** | |  |
| **PREV CLASS**   **NEXT CLASS** | **FRAMES**    **NO FRAMES**     **All Classes** |  |
| SUMMARY: NESTED \| FIELD \| CONSTR \| METHOD | DETAIL: FIELD \| CONSTR \| METHOD |  |


---
